# Magnetic nanocomposites decorated on multiwalled carbon nanotube for removal of Maxilon Blue 5G using the sono-Fenton method

**DOI:** 10.1038/s41598-019-47393-0

**Published:** 2019-07-26

**Authors:** Mehmet Salih Nas, Esra Kuyuldar, Buse Demirkan, Mehmet Harbi Calimli, Ozkan Demirbaş, Fatih Sen

**Affiliations:** 1 Department of Environmental, Faculty of Engineering, University of Igdir, Igdir, Turkey; 2 Sen Research Group, Department of Biochemistry, Faculty of Arts and Science, Dumlupınar University, Evliya Çelebi Campus, 43100 Kütahya, Turkey; 3 Tuzluca Vocational High School, Igdir University, Igdir, Turkey; 4 Department of Chemistry, Faculty of Science and Literature, University of Balikesir, Balikesir, Turkey

**Keywords:** Catalysis, Heterogeneous catalysis

## Abstract

Herein, multiwalled carbon nanotube-based Fe_3_O_4_ nano-adsorbents (Fe_3_O_4_@MWCNT) were synthesized by ultrasonic reduction method. The synthesized nano-adsorbent (Fe_3_O_4_@MWCNT) exhibited efficient sonocatalytic activity to remove Maxilon Blue 5G, a textile dye, and present in a cationic form, in aqueous solution under ultrasonic irradiation. The magnetic nano-adsorbent particles were characterized by high-resolution transmission electron microscopy (HR-TEM), transmission electron microscopy (TEM), Raman spectroscopy and X-ray diffraction (XRD). Some important parameters such as nano-adsorbent dosage, solution pH, initial dye and H_2_O_2_ concentration, reaction time, ultrasonic power and temperature were tested to determine the optimum conditions for the elimination of Maxilon Blue 5G dye. The reusability results showed that Fe_3_O_4_@MWCNT nano-adsorbent has a decrease of about 32.15% in the removal efficiency of Maxilon Blue 5G under ultrasonic irradiation after six times reuse. Additionally, in order to reveal the sufficient kinetic explanation, various experiments were performed at different temperatures and testing three kinetic models like the pseudo-first-order, pseudo-second-order and intraparticle diffusion for removal adsorption process of Maxilon Blue 5G using Fe_3_O_4_@MWCNT nano-adsorbent. The experimental kinetic results revealed that the adsorption process of Maxilon Blue 5G in the aquatic mediums using sono-Fenton method was found to be compatible with the intraparticle diffusion. Using kinetic models and studies, some activation parameters like enthalpy, entropy and Gibbs free energy for the adsorption process were calculated. The activation parameters indicated that Fe_3_O_4_@MWCNT nano-adsorbent could be used as an effective adsorbent for the removal of Maxilon Blue 5G as a textile dye and the adsorption process of Maxilon Blue 5G with Fe_3_O_4_@MWCNT nano-adsorbent is spontaneous.

## Introduction

The dyes are one of the classes of chemical compounds that present as the severe hazards in industrial wastewater^1^. The water pollution caused by dyes poses severe threats to human health. Some diseases such as allergy, dermatitis, skin irritation, cancer, and mutation occur related to dye polluted waters^1–3^. Most of the industrial production facilities, including the textile industry, produce a lot of colored effluents which poses a severe threat to water resources^4,5^. The removal of synthetic wastes from water sources poses a serious threat due to the high dye content and low biodegradability compared to other dyestuffs^6,7^. It is essential to remove organic substances from water sources for a sustainable environment^8^. Many studies have been performed to develop effective solutions for the removal of toxic chemical substances from organic dyes^9–12^. Recently, researchers have effectively utilized different methods based on oxidation techniques to remove toxic organic pollutants^13,14^. Efforts are being made intensively on suitable and efficient technological techniques for the removal of pollutants. One of these methods is the ultrasonic and the Fenton process, which contains an oxidation process^15^. In heterogeneous Fenton-like processes, OH^−^ radicals are formed as Fe^3+^ ions and are converted into Fe^2+^ ions (Reactions 1, 2). Another way to obtain OH^−^ radicals is a fracturing water molecule by sending ultrasound waves (eq. (3)) through cavitation phenomena processes^15^. Hydrogen peroxide could be released by OH^−^ radicals under the influence of ultrasonic radiation in a solution medium (eq. (4))^13–17^.1234

There are some cavitation phenomena such as microbubble formation, precipitation, as well as pressure due to the temperature factor under the ultrasonic wave in the solution environment^18,19^. Furthermore, removal of organic materials by ultrasonic wave method is limited; because a long reaction process is required^20^. Generally, iron-containing nano-adsorbent can be exceeded by integrating the Fenton-like application process to eliminate this obstacle^15^. There are also some disadvantages due to the problems such as the removal of the nano-adsorbent from the wastewater and the accumulation of Fe^+3^ ions in the environment. To overcome this problem, the Fenton processes can be addressed by resorting to heterogeneous catalytic applications^21,22^. Recently, researchers have been interested in the use of particles as catalysts^23–32^. Especially, magnetic nanoparticles provides an opportunity to remove dyes from water sources using nano-adsorbents which have an external magnetic field in heterogeneous Fenton systems^33,34^. This will allow for quick, efficient and easy separation of the magnetic nanoparticles from the water sources^33^. For this purpose, in this study, Fe_3_O_4_@MWCNT were synthesized and used as a nano-adsorbent and not only exhibited a high sonocatalytic activity but also high stability, reusability, and easy application for the removal of Maxilon Blue 5G in aquatic mediums. Fe_3_O_4_@MWCNT nano-adsorbent combined with sono Fenton technique is a non-toxic, cheap and effective solution for the removal of Maxilon Blue 5G from the solution medium. Fe_3_O_4_@MWCNT is an effective example of a new magnetic nano-adsorbent in the sonocatalytic removal of the dye material by the Fenton-like process method. In this study, various parameters such as initial dye concentration, efficient of absorbent, pH, H_2_O_2_ concentration and ultrasonic power (US) were investigated under specific standard parameters. Moreover, the reaction mechanism and the parameters of the thermodynamic function were also studied.

## Experimental

### Materials and methods

FeCl_2_.4H_2_O, FeCl_3_.6H_2_O, Potassium permanganate (KMnO_4_), dimethylformamide, ethanol, hydrogen peroxide (H_2_O_2_), NaOH, sulfuric acid (H_2_SO_4_), sodium nitrate (NaNO_3_), 1,2-tetradecanediol, acetone, and hexanes were purchased from Sigma-Aldrich. Additionally, the natural carbon nanotube chips were supplied from Alfa-Aesar@ company. Experimental studies were carried out with ultrasonic tip sonicator (Bandelin, 40 kHz, 650 W).

### Synthesis of Fe_3_O_4_@MWCNT nano-adsorbents

The synthesis of Fe_3_O_4_@MWCNT nano-adsorbents was achieved by ultrasonic reduction method. Typically, 0.02 g of FeCl_2_.4H_2_O and 0.06 g of FeCl_3_.6H_2_O were dissolved in 200 mL inert gases purged deonized water. Then 100 mL of 1 M NaOH solution was added into the mixture FeCl_2_.4H_2_O and 0.06 g of FeCl_3_.6H_2_O solutions and they were stirred vigorously until obtaining of the black colloidal suspension of Fe_3_O_4_ nano-adsorbents. The obtained nano-adsorbents solution was mixed with 0.00025 g/mL of MWCNT under ultrasonication. The mixture was continued to stirring for 2 days at room temperature. After the obtaing of Fe_3_O_4_@MWCNT, they were separated by a magnet, and then washed at least 3 times and dried under inert atmosphere.

### Experimental adsorption procedure of Maxilon Blue 5G using Fe_3_O_4_@MWCNT nano-adsorbents under sono-Fenton waves

The samples for the characterization process were prepared by taking a 5 mL solution containing Fe_3_O_4_ and MWCNT. This solution was divided into 8 tubes with 5 × 100 volumes and then centrifuged. The formed precipitates were filtered and dried under inert medium, and then stored for further analysis. The X-ray diffraction (XRD) analysis of Fe_3_O_4_@MWCNT nano-adsorbent was performed using an analytical Empyrean diffractometer capable of X-ray diffraction (Cu K, λ = 1.54056 Å, at 45 kV and 40 Ma). Transmission electron microscopy (TEM) analysis was conducted with a JEOL 200 kV instrument. To take TEM analysis various sample was taken to prepare colloidal slurry and the resulting mixtures were dropped on Cu-TEM grid comprised of carbon. The mean particles size of Fe_3_O_4_@MWCNT nano-adsorbent were calculated counting diameter of regions present in TEM patterns. The resulting solution was mixed using Maxilon Blue 5G and Fe_3_O_4_@MWCNT in the dark condition to ensure adsorption-desorption balance for 15 min. The desired temperature, pH, Maxilon Blue 5G concentration at a given initial concentration of H_2_O_2_ (2 mM) and ultrasonic power were adjusted. 4 ml samples were also taken at regular intervals from the reaction solution medium. Then the wastewater is separated by centrifugation (Sigma 3–30 KS) at 15000 rpm and 10 minutes. The measurements were taken by a UV-Vis spectrometer (Perkin Elmer Lambda 750) to determine the concentration of the Maxilon Blue 5G at 410 nm wavelength. The removal efficiency of the dye material was determined from the equation given below.

$${{\rm{q}}}_{{\rm{t}}}=({{\rm{C}}}_{0}-{{\rm{C}}}_{{\rm{t}}}).{\rm{V}}/{\rm{m}}$$

where, C_0_ and C_t_ (mg/L) are the dye concentration for the initial and specific times at equilibrium, respectively. The reusability tests also conducted for the Fe_3_O_4_@MWCNT nano-adsorbents.

## Results and Discussion

### The chemical and morphological analysis of Fe_3_O_4_@MWCNT nano-adsorbent

**Figure 1:**
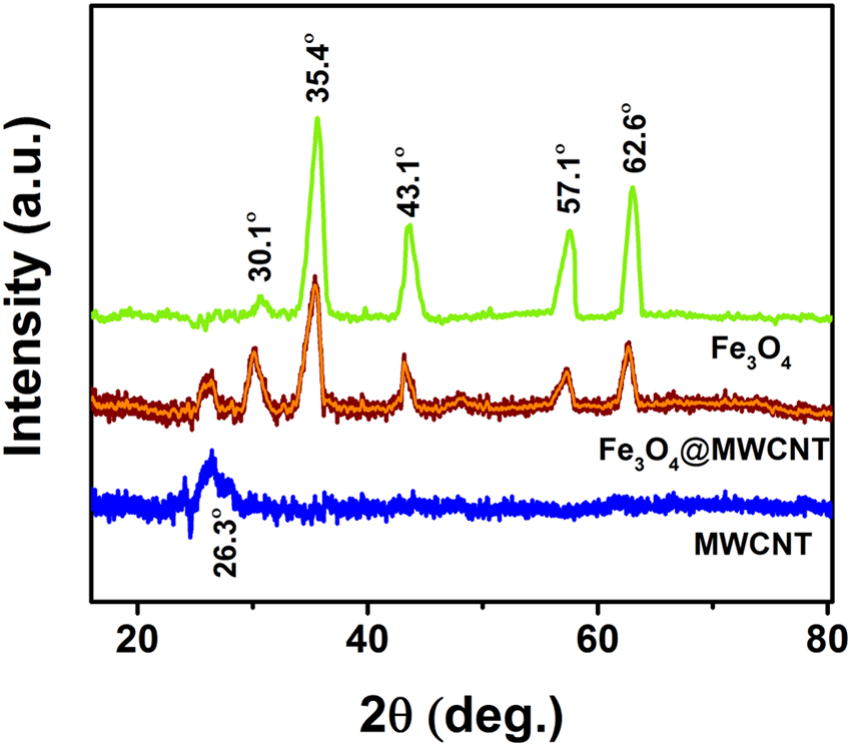
XRD image of MWCNT, Fe_3_O_4_ and Fe_3_O_4_@MWCNT nano-adsorbents.

In order to reveal the crystalline structure of the prepared Fe_3_O_4_@MWCNT nano-adsorbent, XRD analysis has been conducted. The XRD results for Fe_3_O_4_@MWCNT nano-adsorbent are given in Fig. 1 and some distinct peaks at about 2θ = 26.3°, 30.1°, 35.4°, 57,1°, and 62.6° are attributed to 220, 311, 400, 511 crystal plane, respectively. Some evident peak which are main peaks of are intensified at 2θ = 35.4°. The crystalline structure of Fe_3_O_4_@MWCNT nano-adsorbent is found to be a cubic system. Further, the crystalline size of the prepared nano-adsorbent was calculated as 3.57 nm which is very close to the value found in TEM analysis. XRD analysis also indicated that no other impurities were observed except Fe_3_O_4_ present in MWCNT (002, 2θ = 26.3°) samples. These result showed that the structure of Fe_3_O_4_@MWCNT nano-adsorbent is crystalline and pure.

**Figure 2:**
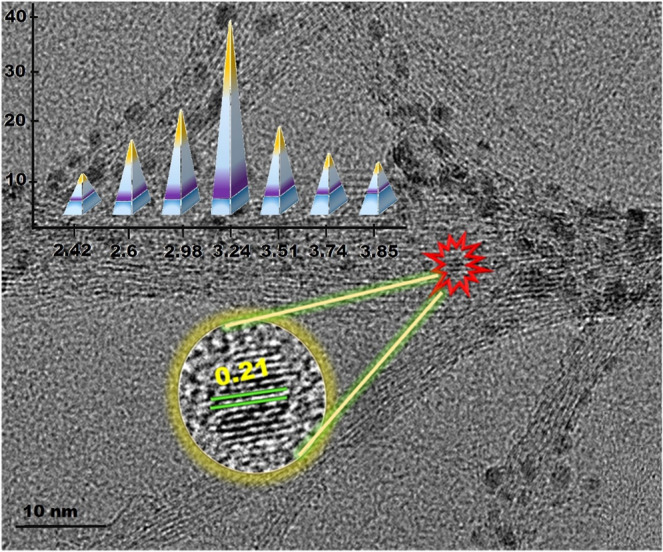
Transmission electron microscopy image, high-resolution transmission electron microscopy image, particle size histogram of magnetic Fe_3_O_4_@MWCNT nano-adsorbent.

TEM and HR-TEM analysis were performed to determine the structural properties of the Fe_3_O_4_@MWCNT catalyst. As can be seen from Fig. 2, the mean particle size of the monodisperse Fe_3_O_4_@MWCNT nanoparticle was 3.24 ± 0.61 nm which is in good agreement with XRD results. In addition, Fig. 2 shows a uniform distribution of Fe_3_O_4_ over the MWCNT without any agglomeration. HR-TEM image also shows that the atomic lattice fringe of Fe_3_O_4_@MWCNT nanoparticle is consistent with the literature data (0.21 nm)^4^. TEM image of Fe_3_O_4_ is also given in Fig. S1.

**Figure 3:**
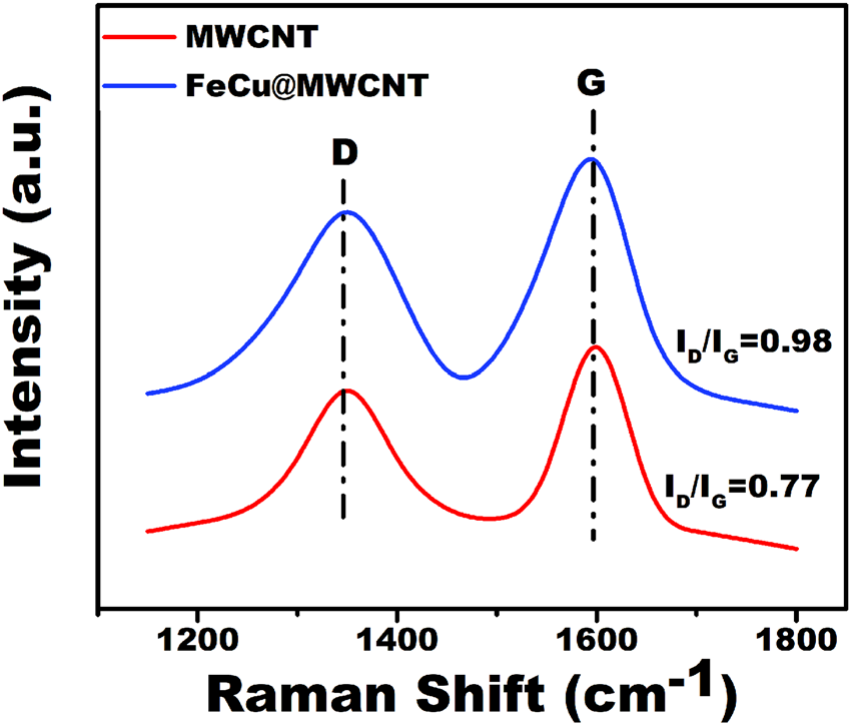
Micro-Raman patterns of Fe_3_O_4_@MWCNTnanocomposites (D band of MWCNT near 1340.5 cm^−1^).

For further structural analysis of synthesized Fe_3_O_4_@MWCNT nano-adsorbent, Raman^35^ spectroscopic analyses were carried out. Raman spectroscopic analysis (given in Fig. 3) of Fe_3_O_4_@MWCNT nano-adsorbent, further details of the structure of Fe_3_O_4_@MWCNT nano-adsorbent were revealed in Fig. 3. The modification and structural disorders were controlled by comparing the density ratios of G and D bands. ID/IG ratios for MWCNT and Fe_3_O_4_@MWCNT were found to be 0.77 and 0.98, respectively. The findings of Raman spectroscopy showed that MWCNT were functionalized with Fe_3_O_4_^36–38^. D band in 1340 cm^−1^ region and G band in 1600cm^−1^ region were observed in Raman spectra of the prepared materials. The vibrations created by carbon on the basal plane and the E_2g_ mode form the G band. D and G bands are formed due to the Raman mechanism in double resonance structure. These bands are directly related to lattice structure and particle size [26, 27]. The ratio obtained from the bands D and G (I_D_/I_G_) is inversely proportional to the size of the crystalline structure of carbon. The I_D_/I_G_ ratios of the Fe_3_O_4_ nanoparticles supported by MWCNT are very high. Raman spectrum of Fe_3_O_4_ is also given in Fig. S2.

### The effect of experimental conditions on the removal of Maxilon Blue 5G using Fe_3_O_4_@MWCNT nano-adsorbents under ultrasonic waves

**Figure 4:**
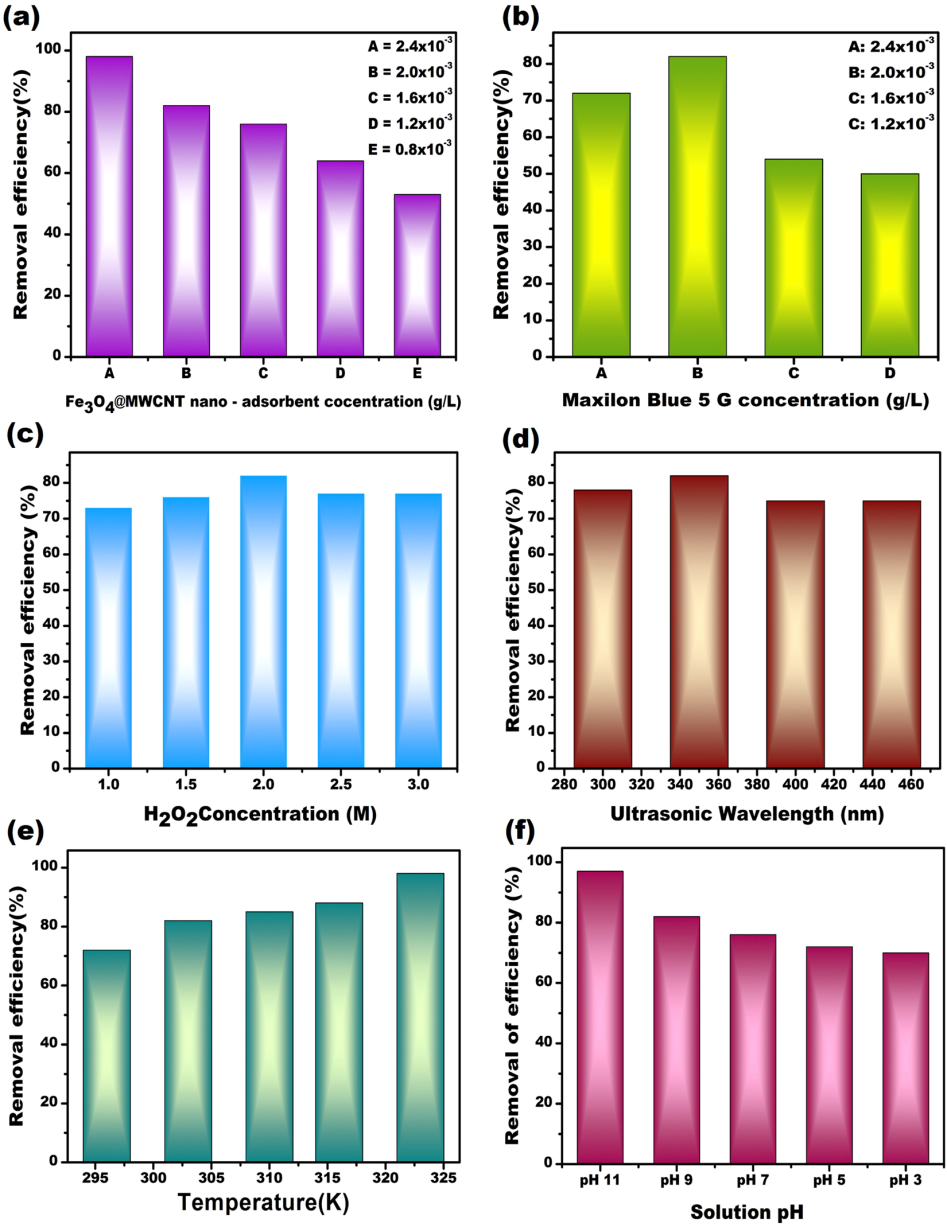
The removal efficiency of Maxilon Blue 5G using Fe_3_O_4_@MWCNT nano-adsorbents at different reaction mediums. (**a**) Fe_3_O_4_@MWCNT Maxilon nano-adsorbent concentrations, (**b**) Maxilon Blue 5G concentrations, (**c**) Ultrasonic wavelength, (**d**) H_2_O_2_ concentrations, (**e**) Temperatures, (**f**) Solution pH. Maxilon Blue 5G = 20 g L^−1^, [Fe_3_O_4_@MWCNT] = 0.020 g L^−1^, [H_2_O_2_] = 2 mM, pH = 9, time = 120 min).

In order to compare the effects of experimental conditions on the removal of Maxilon Blue 5G by Fe_3_O_4_@MWCNT nano-adsorbent, various conditions such as different nano-adsorbent and dye concentrations, ultrasonic wavelength, H_2_O_2_ concentrations, temperatures, and pH were examined and the experimental results achieved at different conditions are given in Fig. 4.

#### The effects of Fe_3_O_4_@MWCNT nano-adsorbent concentrations on the removal efficiency

One of the most effective parameters for removing of Maxilon Blue 5G is the amount of nano-adsorbent concentrations. The nano-adsorbent effects were analyzed at pH of 9 for 120 minutes in solutions containing 2 mM H_2_O_2_. As shown in Fig. 4(a), the increase in the dosage of Fe_3_O_4_@MWCNT magnetic nano-adsorbent was found to be effective for removing Maxilon Blue 5G. As shown in Fig. 4(a), the extraction yield of Maxilon Blue 5G was found to be the highest for the 0.0024 g L^−1^ Fe_3_O_4_@MWCNT magnetic nano-adsorbent dose (about with %98 yield). It can be related to an increase in the number of active catalytic sites due to the increase in the amount of magnetic nano-adsorbent Fe_3_O_4_@MWCNT, and therefore, more reactive radicals can be produced. Furthermore, a larger increase in the amount of magnetic nano-adsorbent particles results in a decrease in the efficiency of Maxilon Blue 5G removals in sono adsorption systems. In this case, the addition of the nano-adsorbent may have a cleaning effect on the %OH^−^ radicals, resulting in a reduction of the Maxilon Blue 5G removal efficiency in the solution medium^15^. Another reason is that in sonocatalytic heterogeneous systems, excessive screening quantification of ultrasonic waves by the magnetic nano-adsorbent particle prevents the same amount of ultrasonic energy from being absorbed^39^. The highest removing amount of the Maxilon Blue 5G by the using of Fe_3_O_4_@MWCNT was also detected, as seen in Fig. 4(a).

#### The effects of Maxilon Blue 5G concentrations on the removal efficiency

To investigate the effect of Maxilon Blue 5G dye concentration, some experiments were conducted at different initial concentrations of Maxilon Blue 5G at constant parameters such as 2 mM of H_2_O_2_ concentration, 303 K temperature, and pH of 9. By increasing the dye concentration of Maxilon Blue 5G from 0.0012 to 0.0024 g. L^−1^ in the sono adsorption process, the efficiency of the Maxilon Blue 5G removals increased from 50.2% to 82.1% within 120 min (Fig. 4(b)). The amount of adsorbed dye on the surface of the magnetic nano-adsorbent material is increased when the amount of dye in the solution medium increased, and this prevents absorption of energy produced due to acoustic cavitation by nano-adsorbent particles^40^. Hence, the percentage of OH^−^ radicals and removing dye capacity will result in a decrease. It can be explained that the removal efficiency of intermediates, which is particularly evident as a result of the interaction of OH^−^ molecules with dye molecules, can be reduced^39^. Also, it blocks active areas on the surface of Fe_3_O_4_@MWCNT as a result of high dye concentration in the solution medium. In this case, it causes the minimal growth of the OH radicals and thus, results in lower Maxilon Blue 5G removal efficiency. Besides, the nitrogen adsorption and desorption isotherms of Fe_3_O_4_@MWCNT nano-adsorbents are also given in Fig. S3 in order to explain the higher efficiency of prepared nano-adsorbents. The analysis was performed by evaluating the hysteresis curve of nitrogen adsorption and desorption at isothermal conditions. According to the analysis, it is known that the Fe_3_O_4_/MWCNT nanocomposites have a large surface area, namely 335 m^2^/g. Such large area would improve the performance of the adsorption property of the nanocomposites and very appropriate for removal of maxilon 5G.

#### The effects of H_2_O_2_ concentrations, ultrasonic wavelength, temperatures, and solution pH on the removal of Maxilon Blue 5G from aqueous medium

Figure 4 also shows the effects of H_2_O_2_ concentrations (Fig. 4c), ultrasonic wavelength (Fig. 4d), temperatures (Fig. 4e), and solution pH (Fig. 4f) on the removal Maxilon Blue 5G from aqueous medium. In this study, the effect of H_2_O_2_ concentration on the elimination of the dye in the solution medium was checked. In heterogeneous Fenton-like systems, the concentration of H_2_O_2_ has a positive effect on the increase of active radicals^41^. To investigate the effect of H_2_O_2_ at different concentrations, experiments were conducted at the constant parameters such as 0.02 g L^−1^ Fe_3_O_4_@MWCNT, pH of 9 in aqueous solution and 120 minutes of reaction time. The experimental results showed that the removal efficiency of Maxilon Blue 5G was highest at 2 M concentration of H_2_O_2_ as shown in Fig. 4c. This situation is considered due to the increase in OH^−^ released to the solution environment. The efficiency of dye elimination decreased at higher hydrogen peroxide (H_2_O_2_) concentrations. This is because the hydrogen peroxide (H_2_O_2_) added after a certain point in the sonocatalytic heterogeneous processes was found to interfere with the interaction between the surface of the nano-adsorbent material and the dye material. As stated in eqs (5) and (6), excessive concentration of hydrogen peroxide in the solution medium can induce OH^−^ radical scavenging effect^42^ and cause reduction of radicals required for oxidation^43,44^.56

One of the essential parameters for the removal of Maxilon Blue 5G dye is the amount of ultrasonic power. To determine the effect of the ultrasonic power, the same constant parameter conditions were prepared with 20 mg L^−1^ of Fe_3_O_4_@MWCNT, pH of 9 and 2 mM H_2_O_2_ concentration. As indicated in Fig. 4(d), the ultrasonic power effect was seen to be more effective in removal efficiency from 350 W to 450 W. It can be explained this situation on two different mechanisms. Firstly, the increase in ultrasound power increased dissolution turbulence. This leading to the higher release of reactive radicals and an increase in the mass transfer rate of Maxilon Blue 5G, which positively contributed to the reduction in the number of by-products present throughout the nano-adsorbent surface^13^. Secondly, the cleaning of the ultrasonic beam responded positively to the increase in power. It is thought that the increase of ultrasonic irradiation causes further expands of active fields on the surface of the magnetic nano-adsorbent^13,45^. In can be concluded that the increase in ultrasonic power results in a further increase of reactive radicals. In order to determine the optimum temperature for the adsorption of Maxilon Blue 5G on Fe_3_O_4_@MWCNT, a set of experiments at different temperatures ranging 296–323 K was performed. The results of the experiments conducted at different temperatures are given in Fig. 4e. The optimum temperature on the adsorption process was found to be 323 K. Increasing temperature effects the interaction of particle, and that increasing interactions increased the adsorption of Maxilon Blue 5G. additionally, the volumes of pores on the adsorbent is increased with increasing temperature^12^. These results affected positively the adsorption amount of Maxilon Blue 5G. The effect of pH solution is also a very crucial parameter in the adsorption process to gain the properties of materials investigated under ultrasonic wave iridations^41,42^. The results for pH effects of the solution containing 0.002 g.L^−1^ Fe_3_O_4_@MWCNT magnetic nanomaterials and 20 mg L^−1^ Maxilon Blue 5G in 120 minutes to remove the Maxilon Blue 5G are given in Fig. 4(f). The highest yield was obtained at a pH of 11. These might be explained by two reasons. The situation of the surface of nano-adsorbent affects the values of pH, and it can be explained according to the zero-load point of Fe_3_O_4_@MWCNT. The zero-load point of the Fe_3_O_4_@MWCNT magnetic nano-adsorbent was determined to be 6.8 by the method specified in the literature^46^. When the pH of the solution lower than the zero-load point of the Fe_3_O_4_@MWCNT magnetic nano-adsorbent, the surface of the nano-adsorbent material is protonated. Similarly, the surface of the nano-adsorbent is deprotonated when a higher pH value applied^47^. For this reason, the cationic dye can be adsorbed onto the Fe_3_O_4_@MWCNT nano-adsorbent, and the surface binding domains of the nano-adsorbent material are affected. Therefore, the ionic state of the Maxilon Blue 5G molecule has great importance. At low pH, the nano-adsorbent surface charge is positively charged and H^+^ the ions encounter an impulsive force effectively with the Maxilon Blue 5G cations, thus causing a reduction in the amount of adsorbed dye. At higher pH values, the magnetic nano-adsorbent particle increases the negatively charged density. By this way, the electrostatic attraction forces between the support material and the cationic dye can be increased^48,49^. Besides, as shown in Table S1, the iron ion concentration in the solution medium increased at high pH. This relates to both the absolute concentration of dissolved iron and the increased dissociation of OH^−^ radicals of H_2_O_2_ molecules in the heterogeneous sono-Fenton process^50^. As a result, the presence of % OH^−^ radicals also significantly affected the electrostatic attraction between the nano-adsorbent and the dye. The most efficient removal was achieved at an optimum pH value of 11 (Fig. 4(f)).

#### The comparisons of some parameters studied for Maxilon Blue 5G removal using Fe_3_O_4_@MWCNT nano-adsorbent and their reusability efficiency

**Figure 5:**
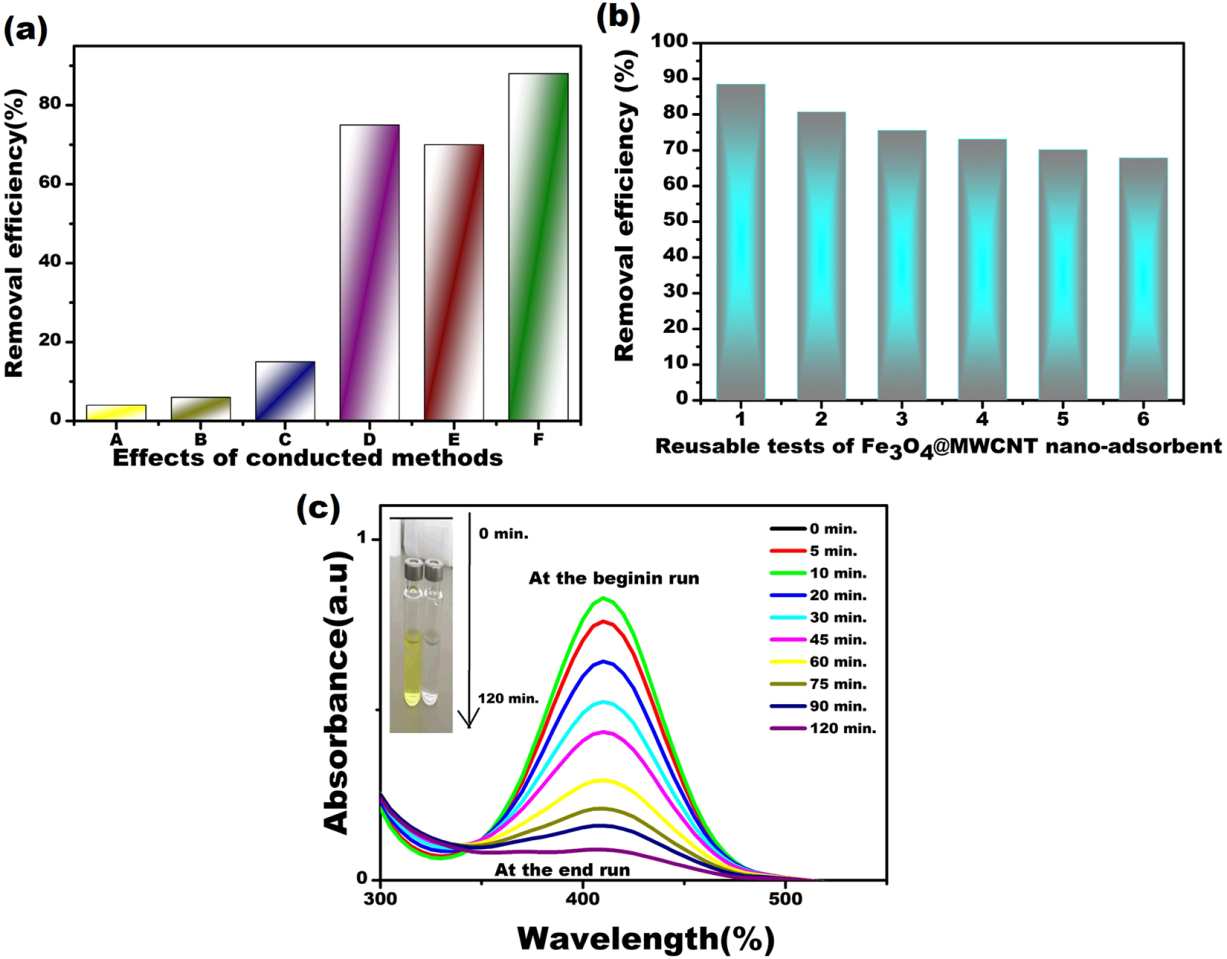
(**a**) The effects of different experimental conditions on the removal of Fe_3_O_4_@MWCNT Ultrasonic waves (A), H_2_O_2_ concentrations (B), Fe_3_O_4_@MWCNT concentrations (C), Fe_3_O_4_@MWCNT/H_2_O_2_ (D), Fe_3_O_4_@MWCNT/Ultrasonic wavelength (E), Fe_3_O_4_@MWCNT/Ultrasonic wavelength/H_2_O_2_ (F). (**b**) Reusability tests of Fe_3_O_4_@MWCNTnano-adsorbent in the Maxilon Blue 5G aqueous solution. (**c)** Absorbance change by time: Maxilon Blue 5G aqueous solution containing Fe_3_O_4_@MWCNT adsorbent at 300–500 nm. [Maxilon Blue 5G] = 20 mg L^−1^, [Fe_3_O_4_@MWCNT] = 0.020 g L^−1^, [H_2_O_2_] = 2 mM, UP = 350 W, pH = 9 and time = 120 min).

The experiments of removal of Maxilon Blue 5G conducted at different parameters were carried out at pH of 9 with 10 mgL^−1^ of Fe_3_O_4_@MWCNT nano-adsorbent; the results of these experiments are given in Fig. 5a. As indicated in Fig. 5a, the efficiency of Maxilon Blue 5G removal was determined to be approximately 3.75% and 5.72% after operating the system for 120 minutes using ultrasonic wave and H_2_O_2,_ respectively. The data obtained under these experimental conditions show how stable Maxilon Blue 5G is. The efficiency of Fe_3_O_4_@MWCNT magnetic nano-adsorbent in removing the Maxilon Blue 5G dye was not nearly at the desired level. Among the different compositions of the prepared nano-adsorbents as shown in Fig. 5a, Fe_3_O_4_@MWCNT/H_2_O_2_ nano-adsorbent exhibited a best efficiency for the removal of Maxilon Blue 5G compared to the others. As seen in Fig. 5a, the conversion of H_2_O_2_ to free OH radicals in the Fe_3_O_4_@MWCNT/H_2_O_2_ system positively increased the oxidation process in experimental studies (6). As stated in the eq. (1) the interaction between active sites of the Fe_3_O_4_@MWCNT surface and H_2_O_2_ supplied a positive increase in the percentage of OH^−^ radicals. In the US/Fe_3_O_4_@MWCNT/H_2_O_2_ working process, the magnetic nano-adsorbents interacted with ultrasonic waves and produced a higher contact area of magnetic nano-adsorbent as a support material^50^. The heterogeneous catalytic efficiency has been enhanced because of the formation of cavitation microbubbles and their collapse on the surface of the magnetic nano-adsorbent^51,52^. The amount of iron and OH^−^ radicals present on the surface of the nanoparticles increased the efficiency of the Fenton-like process depend on eqs ((1) and (2))^9^.

Another most critical parameters of nano-adsorbents in the removal of dye studies is the reusability tests conducted to investigate the stability of the synthesized materials^10^. The stability of Fe_3_O_4_@MWCNT magnetic nano-adsorbents was investigated reusability in 6 sequential reuse at fixed parameters with 0.020 g L^−1^ nano-adsorbent, 20 mg L^−1^ Maxilon Blue 5G dye, 2 mM H_2_O_2_, pH of 9, 120 minutes. The magnetic nano-adsorbents were magnetically separated from the treated solution after each treatment and washed using ultrapure water, dried and reused for subsequent work (Fig. 5b). As shown in Fig. 5b, six successive dye-extraction yields were obtained in % 88.51, 80.72, 75.56, 73.09, 70.16, and %67.85 respectively. The obtained data in Fig. 5b demonstrate the reusability of Fe_3_O_4_@MWCNT magnetic nano-adsorbents for treatment of wastewater. TEM image of used nano-adsorbents was obtained as shown in Fig. S4 and it was seen that some of the particles was agglomerated which results in the decrease of the reusability efficiency. We have also checked the content of the catalyst with the help of ICP (Inductively Coupled Plasma spectroscopy) in order to see whether there is any leaching in nanocomposite or not and we have seen that there was no leaching in nanocomposite.

**Figure 6:**
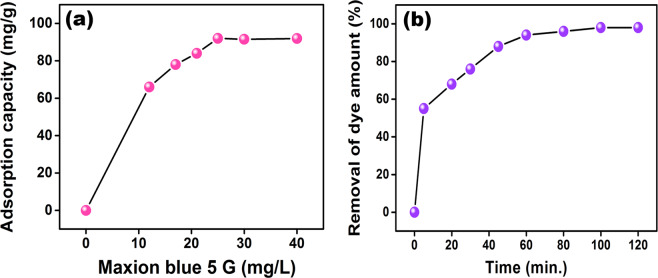
The adsorption capacities for Maxilon Blue 5G at different concentrations by the Fe_3_O_4_@MWCNT nanoparticles, the initial concentration of Maxilon Blue 5G is 5–40 mg/L and the adsorption time is 120 min; (**b**) Percentage removal for Maxilon Blue 5G by the Fe_3_O_4_@MWCNT nanoparticles.

Also, the recycling of the Fe_3_O_4_@MWCNT nano-adsorbent film from water is crucial to prevent further contamination during wastewater treatment in industrial applications because the nanoadsorbent can be easily removed from the water using magnetic force. The absorbance changes of Maxilon Blue 5G containing Fe_3_O_4_@MWCNT adsorbent at 300–500 nm (Maxilon Blue 5G of 20 mg L^−1^, Fe_3_O_4_@MWCNT of 0.020 g L^−1^, H_2_O_2_ of 2 mM, UP of 350 W, pH of 9) for 120 min are shown in Fig. 5c. The maximum absorbance value was obtained as >90%. Figure 5c shows the initial and latest solution color and absorbance values for Maxilon Blue 5G aqueous solution containing Fe_3_O_4_@MWCNT adsorbent. As shown in this figure, Maxilon Blue 5G lost its color during the adsorption process after interaction with Fe_3_O_4_@MWCNT adsorbent.Fig. 6a,b shows adsorption capacity of Fe_3_O_4_ nano-adsorbent and its adsorption amount from aqueous mediums. As seen in Fig. 6a the initial concentration of Maxilon Blue 5G was investigated in concentrations ranging 5–40 mg/L for 120 min. The highest adsorption value of Maxilon Blue 5G on Fe_3_O_4_@MWCNT nano-adsorbents was obtained as 25 mg/L. Figure 6b shows that the maximum adsorption capacity of Maxilon Blue 5G on Fe_3_O_4_@MWCNT nano-adsorbent was reached in almost 60 min. and after this time the adsorption process has reached the equilibrium. According to obtained data, the Fe_3_O_4_@MWCNT magnetic nano-adsorbents proved to be a very effective nano-adsorbent to remove Maxilon Blue 5G under different parameters.

### Kinetic parameters and their calculation for sono -Fenton-like method

Three models were used to find the sufficient kinetic model for the adsorption of Maxilon Blue 5G using Fe_3_O_4_@MWCNT magnetic nano-adsorbent through heterogeneous under the ultrasonic irradiations. The equations used in the calculation to determine the sufficient model are given formulas^53^, where the t is time (min.), k_i_ is adsorption rate constant, q_e_, and q_t_ are the initial and final concentration (mol. g^−1^) of Maxilon Blue 5G dye, respectively. The calculation results obtained from the models are seen in Table S1. Equations of 7, 8 are the first order and second-order models, respectively^54^. In eq. (8); time is t, k_2_ is a constant rate at the adsorption equilibrium, the Maxilon Blue 5G amount is q_e_ (mol. min^−1^). Equation (9) was used to calculate halftime of adsorption process for Maxilon Blue 5G with Fe_3_O_4_@MWCNT nano-adsorbent under ultrasonic wave irradiations. The eq. (10) is used to calculate the initial adsorption rate, h (mol/(g min) and in the values of t_1/2_, k_2_ and q_e_ were calculated and given in Table S1. The initial rate of the intraparticle diffusion is calculated using eq. (11)^55^.

7 $$\mathrm{ln}({{\rm{q}}}_{{\rm{e}}}-{{\rm{q}}}_{{\rm{t}}})=\,\mathrm{ln}\,{{\rm{q}}}_{{\rm{e}}}-{{\rm{k}}}_{{\rm{i}}}{\rm{t}}$$

8 $$\frac{t}{qe}=\frac{1}{{k}_{2}{q}_{e}^{2}}+\frac{1}{{q}_{e}}t$$

9 $${t}_{1/2}=\frac{1}{{k}_{2}{q}_{e}}$$

10 $$h={k}_{2}{q}_{e}$$

11 $${q}_{t}={k}_{int}{t}_{1/2}+C$$

Table S2 shows k_int_ values (mg (g min^−1/2^)^−1^ calculated from the intra-particle diffusion model. The studies in the literature revealed that the slopes between q_t_ and t_1/2_ are multilinear; the graph of q_t_ with t_1/2_ is multilinear^52^. In the adsorption process of Maxilon Blue 5G containing Fe_3_O_4_@MWCNT nano-adsorbent, the first stage of the adsorption process is compatible with the intraparticle. In Fig. 6 the first portion curve exhibited the boundary layer effect in the adsorption process and the second curve shows the effect of the intraparticle and diffusion in pores. Table S2 shows the k_int1,2_ values. The first plot values are so high, and these values are not sufficient for the first stage. k_int2_ is used in the intraparticle diffusion and is compatible with the second linear plot (mol.g.mol^−1/2^)^56^. The ln [(C_t_.Co^−1^)^−1^(1 + mK)] is used for obtaining R^12^ and R_2_^2^ calculated values^57^ and its values are seen in Table S2. The model of mass transfer equations values with particle distribution equations is not appropriates for the adsorption of Maxilon Blue 5G on Fe_3_O_4_@MWCNT nano-adsorbent.

### The calculation of thermodynamic parameters

To calculate the activation parameters for the adsorption of Maxilon Blue 5G using Fe_3_O_4_@MWCNT nano-adsorbent from the aqueous medium, Arrhenius Equation (eq. 12) and k_2_ values were used as shown in Fig. S5. In eq. (12), R is gas constant (J.K^−1^.mol^−1^), and T is temperature (K). The activation energy of Maxilon Blue 5G using Fe_3_O_4_ nano-adsorbent was found to be 27 k J.mol^−1^. Generally, the adsorption process having enthalpies less than 40 k J.mol^−1^ was considered as physical interactions. Vice versa the adsorption processes which having enthalpies higher than 40 k J.mol^−1^ was considered as chemical processes^58^. The following eqs (12 and 13) are used to calculate the other activation parameters^57^.

12 $$ln{k}_{2}=lnA-\frac{Ea}{R.T}$$

13 $$ln({k}_{2}/T)=ln({\rm{kb}}/h)+\frac{{\rm{\Delta }}S}{R}-\frac{{\rm{\Delta }}H}{RT}$$

Where; enthalpy, entropy, adsorption rate, Boltzmann constant, gas constant and Planck constant (6.6261.10^−34^ Js) are ∆H, ∆S, k_2_, k_b_, R (1.3807.10^−23^ JK^−1^) and h, respectively.

The results for these activation parameters and kinetic data are given in Table S3. Fig. S5a,b shows Arrhenius plots for calculations the adsorption parameters for removal Maxilon Blue 5B dye. The value of ∆S (entropy change) was founded to be −94 J.K.mol^−1^. This value indicates that the Maxilon Blue 5G dye was distributed regularly on the Fe_3_O_4_@MWCNT magnetic nano-adsorbent. The results also revealed that the adsorption mechanism for Maxilon Blue 5G dye containing Fe_3_O_4_@MWCNT magnetic nano-adsorbent occurs spontaneously. It was determined that the sonocatalytic removal of the magnetic nano-adsorbent particle was suitable for Langmuir-Hinshelwood kinetic expression by looking at the obtained regression coefficient (R^2^ = 0.9930). The calculation activation parameters were performed using eq. (14) as given below;

14 $${\rm{\Delta }}{\rm{G}}={\rm{\Delta }}{\rm{H}}-{\rm{T}}.{\rm{\Delta }}{\rm{S}}$$

The calculated values of the adsorption of Maxilon Blue 5G dye on the Fe_3_O_4_@MWCNT surface were given in Table S3.

## Conclusion

In this work, Fe_3_O_4_@MWCNT magnetic nano-adsorbent particles were synthesized by ultrasonic reduction method. The nano-adsorbent particles from the data obtained in experimental studies were found to have extreme sonocatalytic efficiency in eliminating dyes from aqueous medium under ultrasonic condition. It has been proved that Maxilon Blue 5G dye is successfully removed from the aqueous solution by using Fe_3_O_4_@MWCNT as the adsorbent material and with the help of ultrasonication, separately. The experimental process reached a disposal efficiency of %92 at pH of 9 after a 120-minute reaction period. The obtained data showed that OH. radicals play a significant role in the removal of Maxilon Blue 5G dye by the sonocatalytic method in the presence of Fe_3_O_4_@MWCNT magnetic nano-adsorbent. Moreover, the reusability test has shown the stability of Fe_3_O_4_@MWCNT magnetic nano-adsorbents with very high sonocatalytic removal efficiency under optimum conditions. The thermodynamic parameters such as Gibbs free energy (ΔG∗), Ea, ΔH*, and ΔS* were calculated as −61.465, 27.01, 32.325 kJ mol^−1^ and 94.00 J mol^−1^ K^−1^ for removal of Maxilon Blue 5G dye, respectively. According to the calculated values of free Gibbs Energy also shows that the adsorption process occurs spontaneously. It was also determined that the most suitable kinetic model for the adsorption mechanism was intra-particle diffusion models. As a result, the prepared Fe_3_O_4_@MWCNT nano-adsorbent is very effective for the removal of the dyes from industrial wastewater.

## Supplementary information

The related characterization part and supplemantary figures of Magnetic nanocomposites decorated on multiwalled carbon nanotube for removal of Maxilon Blue 5G using the sono-Fenton method.
