# Supplementary material for: Magnetic nanocomposites decorated on multiwalled carbon nanotube for removal of Maxilon Blue 5G using the sono-Fenton method
Source: Sci Rep. 2019 Jul 26;9:10850. doi: 10.1038/s41598-019-47393-0 (PMC6659670; doi:10.1038/s41598-019-47393-0)
Supplement: Supplementary file 1 — The related characterization part and supplemantary figures of Magnetic nanocomposites decorated on multiwalled carbon nanotube for removal of Maxilon Blue 5G using the sono-Fenton method. [file 41598_2019_47393_MOESM1_ESM.docx]

**SUPPORTING INFORMATION**

**Magnetic nanocomposites decorated on multiwalled carbon nanotube for removal of Maxilon Blue 5G using the sono-Fenton method**

Mehmet Salih Nas^a*^, Esra Kuyuldar^b^, Buse Demirkan^b^, Mehmet Harbi Calimli^c^, Ozkan Demirbaş^a^, Fatih Sen^b*^

^a^Department of Chemistry, Faculty of Science and Literature, University of Balikesir, Balikesir, Turkey

^b^Sen Research Group, Department of Biochemistry, Faculty of Arts and Science, Dumlupınar

University, Evliya Çelebi Campus, 43100 Kütahya, Turkey

^c^Tuzluca Vocational High School, Igdir University, Igdir, Turkey

^d^Department of Environmental, Faculty of Engineering, University of Igdir, Igdir, Turkey

^*^Corresponding author: fatih.sen@dpu.edu.tr, mehmet.salih.nas@igdir.edu.tr

Tel:90 274 265 20 31 -37 02 Fax:90 274 265 20 56

**Synthesis of Fe_3_O_4_@MWCNT nano-adsorbents**

Typically, 0.02 g of FeCl_2_.4H_2_O and 0.06 g of FeCl_3_.6H_2_O were dissolved in 200 mL N_2_-purged distilled water. Then 100 mL of an aqueous solution of 1 M NaOH was slowly dropped into the mix solution under vigorous stirring. The mixture was continuously stirred for 2-3 h. The black colloidal suspension of Fe_3_O_4_ nano-adsorbents was obtained. Fe_3_O_4_/MWCNT composites were prepared from, 22.0 mL of the black colloidal of Fe_3_O_4_ was slowly dropped in 0.0125 g of MWCNT dispersed in 50 mL of with N_2_ purged distilled water under ultrasonication. The mixture was stirred for 2 days at room temperature. The particles were separated by a permanent magnet, allowing the particles to be washed with N_2_-purged distilled water. The washing procedure was repeated for 3 times and dried under N_2_ atmosphere.


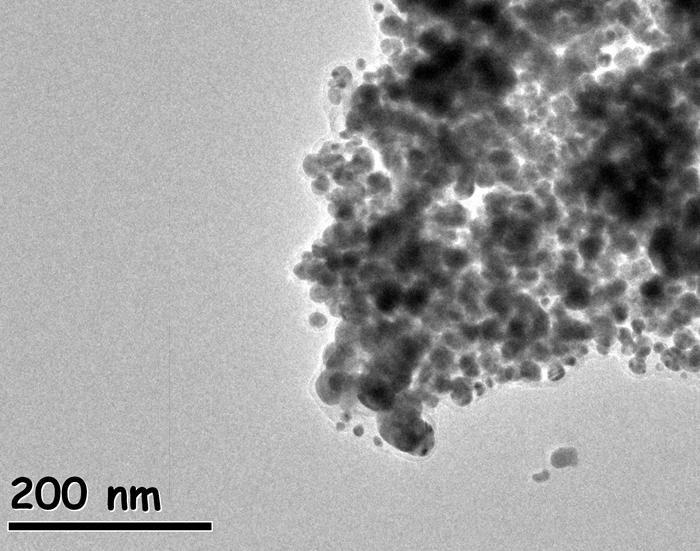


**Fig. S1.** The TEM image of Fe_3_O_4_ nanoparticles

**
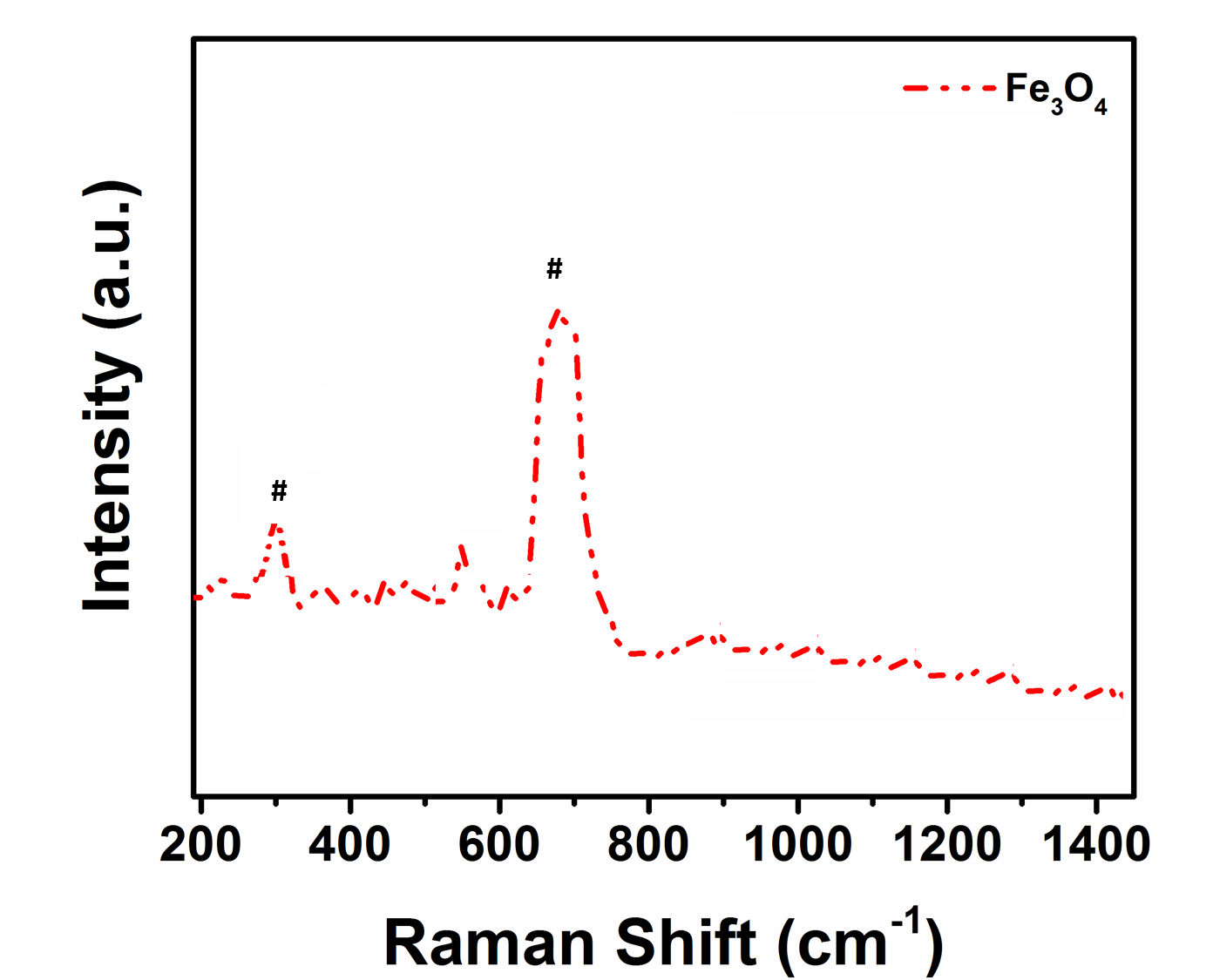
**

**Fig. S2.** The Raman spectrum of Fe_3_O_4_ nanoparticles

**

**

**Fig. S3.** The nitrogen adsorption and desorption isotherms of Fe_3_O_4_@MWCNT nano-adsorbents

**
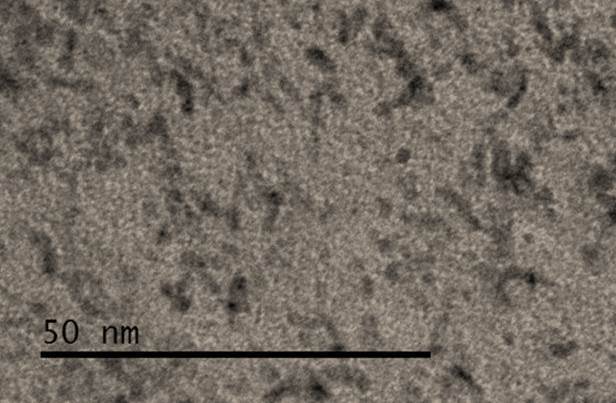
**

**Fig. S4.** The TEM image of Fe_3_O_4_@MWCNT nanoparticles after their 5^th^ reusability performance

**Table S1.** Kinetic data of magnetic Fe_3_O_4_@MWCNT nano-adsorbent for the adsorption of Maxilon Blue 5G.

| T/K | Fe_3_O_4_@MWCNT dosage (g.L^-1^) | PH | US power (W) | MB 5G  g.L^-1^ | [H_2_O_2_]  (mM) | Pseudo second-order R^2^ | q_e_ (cal.) (mg.g^-1^) | q_e_ (exp.) (mg.g^-1^) | k_2_ (g.mg  min^-1^) | R^2^ | h(mol min^-1^g^-1^) | t_1/2_ (min) |
| --- | --- | --- | --- | --- | --- | --- | --- | --- | --- | --- | --- | --- |
| 296 | 0.02 | 9 | 350 | 0.02 | 2 | 0.56 | 0.064 | 0.063 | 0.014 | 0.85 | 0.0008 | 1,250 |
| 303 | 0.02 | 9 | 350 | 0.02 | 2 | 0.65 | 0.0741 | 0.0742 | 0.020 | 0.88 | 0.0014 | 714 |
| 310 | 0.02 | 9 | 350 | 0.02 | 2 | 0.76 | 0.076 | 0.076 | 0.029 |  | 0.0022 | 454 |
| 316 | 0.02 | 9 | 350 | 0.02 | 2 | 0.65 | 0.080 | 0.080 | 0.033 | 0.95 | 0.0026 | 384 |
| 323 | 0.02 | 9 | 350 | 0.02 | 2 | 0.68 | 0.088 | 0.088 | 0.036 | 0.92 | 0.0034 | 294 |
| 303 | 0.02 | 3 | 350 | 0.02 | 2 | 0.75 | 0.058 | 0.058 | 0.016 | 0.90 | 0.0009 | 1,111 |
| 303 | 0.02 | 5 | 350 | 0.02 | 2 | 0.84 | 0.063 | 0.062 | 0.018 | 0.93 | 0.0011 | 909 |
| 303 | 0.02 | 7 | 350 | 0.02 | 2 | 0.76 | 0.068 | 0.068 | 0.019 | 0.96 | 0.0013 | 769 |
| 303 | 0.02 | 11 | 350 | 0.02 | 2 | 0.85 | 0.088 | 0.087 | 0.026 | 0.99 | 0.0023 | 434 |
| 303 | 0.02 | 9 | 350 | 0.02 | 1 | 0.87 | 0.066 | 0.066 | 0.0101 | 0.94 | 0.0007 | 1,428 |
| 303 | 0.02 | 9 | 350 | 0.02 | 1.5 | 0.84 | 0.068 | 0.068 | 0.0189 | 0.92 | 0.0012 | 833 |
| 303 | 0.02 | 9 | 350 | 0.02 | 3.5 | 0.77 | 0.068 | 0.068 | 0.024 | 0.87 | 0.0016 | 625 |
| 303 | 0.02 | 9 | 350 | 0.02 | 3 | 0.79 | 0.068 | 0.067 | 0.030 | 0.95 | 0.0020 | 500 |
| 303 | 0.024 | 9 | 350 | 0.02 | 2 | 0.88 | 0.088 | 0.088 | 0.024 | 0.96 | 0.0021 | 476 |
| 303 | 0.016. | 9 | 350 | 0.02 | 2 | 0.76 | 0.068 | 0.068 | 0.020 | 0.98 | 0.0014 | 714 |
| 303 | 0.012 | 9 | 350 | 0.02 | 2 | 0.85 | 0.056 | 0.057 | 0.021 | 0.88 | 0.0012 | 833 |
| 303 | 0.008 | 9 | 350 | 0.02 | 2 | 0.87 | 0.046 | 0.046 | 0.012 | 0.98 | 0.0005 | 2,000 |
| 303 | 0.02 | 9 | 300 | 0.02 | 2 | 0.84 | 0.071 | 0.071 | 0.011 | 0.92 | 0.0007 | 1,428 |
| 303 | 0.02 | 9 | 400 | 0.02 | 2 | 0.77 | 0.068 | 0.067 | 0.009 | 0.86 | 0.0006 | 1,666 |
| 303 | 0.02 | 9 | 450 | 0.02 | 2 | 0.79 | 0.067 | 0.067 | 0.011 | 0.77 | 0.0007 | 1,428 |
| 303 | 0.02 | 9 | 350 | 0.024 | 2 | 0.88 | 0.063 | 0.062 | 0.011 | 0.90 | 0.0007 | 1,428 |
| 303 | 0.02 | 9 | 350 | 0.016 | 2 | 0.89 | 0.049 | 0.048 | 0.016 | 0.96 | 0.0008 | 1,250 |

**Table S2.** Mechanism Kinetic data calculated for the adsorption of Maxilon Blue 5G using Fe_3_O_4_@MWCNT nano-adsorbent from aquatic mediums.

| Mass transfer | | | | | | Intraparticle diffusion | | | | |
| --- | --- | --- | --- | --- | --- | --- | --- | --- | --- | --- |
| T/K | **Fe_3_O_4_@MWCNT**  **g.L^-1^** | **pH** | **US  power (W)** | **[H2O2]**  **(mM)** | **MB 5G g.L^-1^** | **R^2^** | **kint,1 mg.g^-1^ min^-1/2^** | **R_1_^2^** | \| **kint,_2_ mg.g^-1^ min^-1^** \| \| --- \| | **R_2_^2^** |
| 296 | 0.02 | 9 | 350 | 2 | 0.02 | 0.98 | 0.0035 | 0.99 | 0.0084 | 0.99 |
| 303 | 0.02 | 9 | 350 | 2 | 0.02 | 0.99 | 0.0050 | 0.99 | 0.0086 | 0.99 |
| 310 | 0.02 | 9 | 350 | 2 | 0.02 | 0.99 | 0.0072 | 0.99 | 0.0078 | 0.99 |
| 316 | 0.02 | 9 | 350 | 2 | 0.02 | 0.99 | 0.0079 | 0.99 | 0.0073 | 0.99 |
| 323 | 0.02 | 9 | 350 | 2 | 0.02 | 0.99 | 0.0110 | 0.99 | 0.0065 | 0.94 |
| 303 | 0.02 | 3 | 350 | 2 | 0.02 | 0.98 | 0.0036 | 0.99 | 0.0075 | 0.99 |
| 303 | 0.02 | 5 | 350 | 2 | 0.02 | 0.98 | 0.0045 | 0.98 | 0.0075 | 0.99 |
| 303 | 0.02 | 7 | 350 | 2 | 0.02 | 0.99 | 0.0055 | 0.99 | 0.0075 | 0.99 |
| 303 | 0.02 | 11 | 350 | 2 | 0.02 | 0.98 | 0.0100 | 0.99 | 0.0053 | 0.99 |
| 303 | 0.02 | 9 | 350 | 1 | 0.02 | 0.99 | 0.0037 | 0.99 | 0.0097 | 0.99 |
| 303 | 0.02 | 9 | 350 | 1.5 | 0.02 | 0.99 | 0.0036 | 0.99 | 0.0068 | 0.99 |
| 303 | 0.02 | 9 | 350 | 2.5 | 0.02 | 0.99 | 0.0035 | 0.99 | 0.0068 | 0.99 |
| 303 | 0.02 | 9 | 350 | 3 | 0.02 | 0.99 | 0.0034 | 0.99 | 0.0040 | 0.99 |
| 303 | 0.024 | 9 | 350 | 2 | 0.02 | 0.98 | 0.0094 | 0.98 | 0.0054 | 0.99 |
| 303 | 0.016. | 9 | 350 | 2 | 0.02 | 0.98 | 0.0055 | 0.99 | 0.0076 | 0.99 |
| 303 | 0.012 | 9 | 350 | 2 | 0.02 | 0.99 | 0.0036 | 0.99 | 0.0091 | 0.99 |
| 303 | 0.008 | 9 | 350 | 2 | 0.02 | 0.98 | 0.0025 | 0.98 | 0.0065 | 0.99 |
| 303 | 0.02 | 9 | 300 | 2 | 0.02 | 0.99 | 0.0032 | 0.99 | 0.0054 | 0.99 |
| 303 | 0.02 | 9 | 400 | 2 | 0.02 | 0.99 | 0.0038 | 0.99 | 0.0070 | 0.99 |
| 303 | 0.02 | 9 | 450 | 2 | 0.02 | 0.99 | 0.0054 | 0.99 | 0.0066 | 0.99 |
| 303 | 0.02 | 9 | 350 | 2 | 0.024 | 0.99 | 0.0045 | 0.99 | 0.0074 | 0.99 |

**Table S3.** The activation parameters obtained from the adsorption of Maxilon Blue 5G dye on the Fe_3_O_4_@MWCNT nano-adsorbent.

| Parameters | ΔG | Ea | ΔH | ΔS |
| --- | --- | --- | --- | --- |
| (T/K) | **(kJ/mol)** | **(kJ/mol)** | **(kJ/mol)** | **(j/K.mol)** |
| 296 | -60.149 |  |  |  |
| 303 | -60.807 |  |  |  |
| 310 | -61.465 | 27.01 | -32.325 | 94.00 |
| 316 | -62.029 |  |  |  |
| 323 | -62.687 |  |  |  |

**
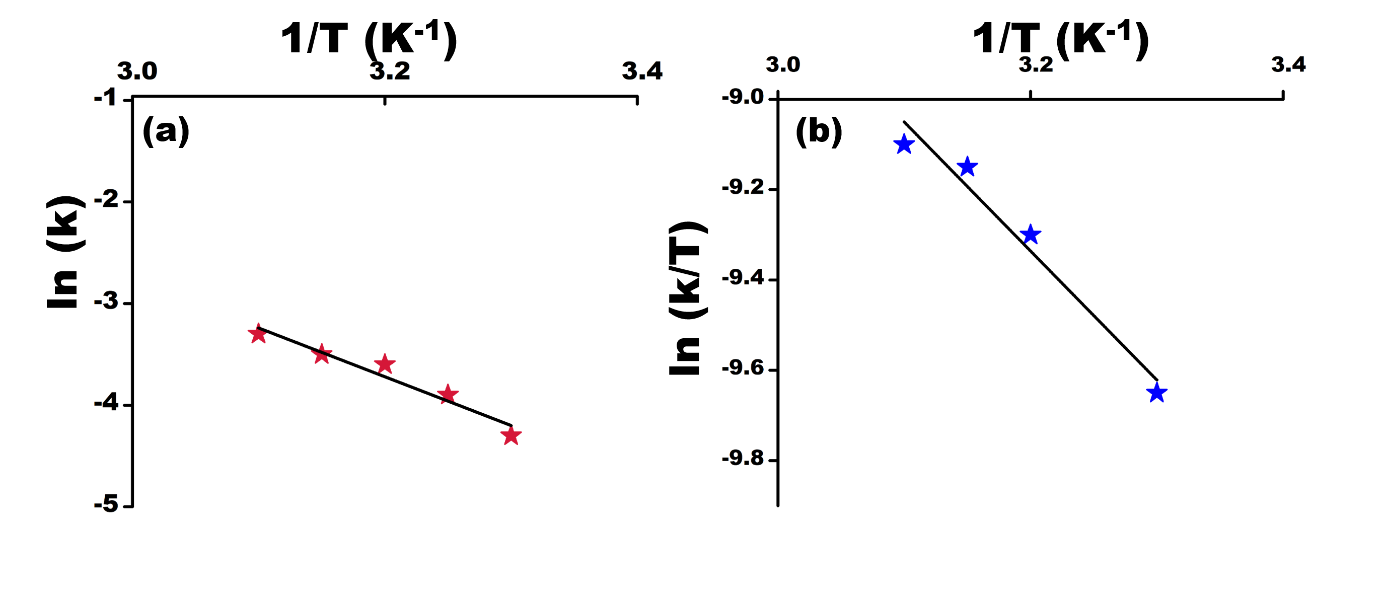
**

**Fig. S5.** (a) Arrhenius plot and (b) thermodynamic function for the removal of Maxilon Blue 5G on Fe_3_O_4_@MWCNT magnetic nano-adsorbent through heterogeneous sono-Fenton-like under the ultrasonic irradiation
